# Supplementary material for: Realistic fisheries management reforms could mitigate the impacts of climate change in most countries
Source: PLoS One. 2020 Mar 5;15(3):e0224347. doi: 10.1371/journal.pone.0224347 (PMC7058327; doi:10.1371/journal.pone.0224347)
Supplement: S1 Table — * See Table S1 in Gaines et al. [19] for earth system models contributing to ensemble mean. Note: RCP 2.6 is not evaluated because it is no longer likely (Raftery et al. 2017). (DOCX) [file pone.0224347.s001.docx]

**Table S1.** Representative Concentration Pathways (RCPs) evaluated in the analysis*.

| **Scenario** | **Projected warming (°C)**** | **Description***** | **Reference** |
| --- | --- | --- | --- |
| RCP 4.5 | 1.8 (1.1-2.6) | Stabilization without overshoot pathway to 4.5 W/m^2^ (~650 ppm CO^2^ eq) at stabilization after 2100 | Clarke et al. 2007; Smith & Wigley 2006; Wise et al. 2009 |
| RCP 6.0 | 2.2 (1.4-3.1) | Stabilization without overshoot pathway to 6.0 W/m^2^ (~850 ppm CO_2_ eq) at stabilization after 2100 | Fujino et al. 2006; Hijioka et al. 2008 |
| RCP 8.5 | 3.7 (2.6-4.8) | Rising radiative forcing pathway leading to 8.5 W/m^2^ (~1370 ppm CO_2_ eq) by 2100 | Riahi et al. 2007 |

* See Table S1 in Gaines et al. (2018) for earth system models contributing to ensemble mean. Note: RCP 2.6 is not evaluated because it is no longer likely (Raftery et al. 2017).

** Projected (2081-2100) mean and likely range for increases in global air temperatures relative to 1986–2005; from Table 2.1 in IPCC AR5.

*** Based on Table 2 in van Vuuren et al. 2011.
