# Supplementary figures and images for: Realistic fisheries management reforms could mitigate the impacts of climate change in most countries
Source: PLoS One. 2020 Mar 5;15(3):e0224347. doi: 10.1371/journal.pone.0224347 (PMC7058327; doi:10.1371/journal.pone.0224347)

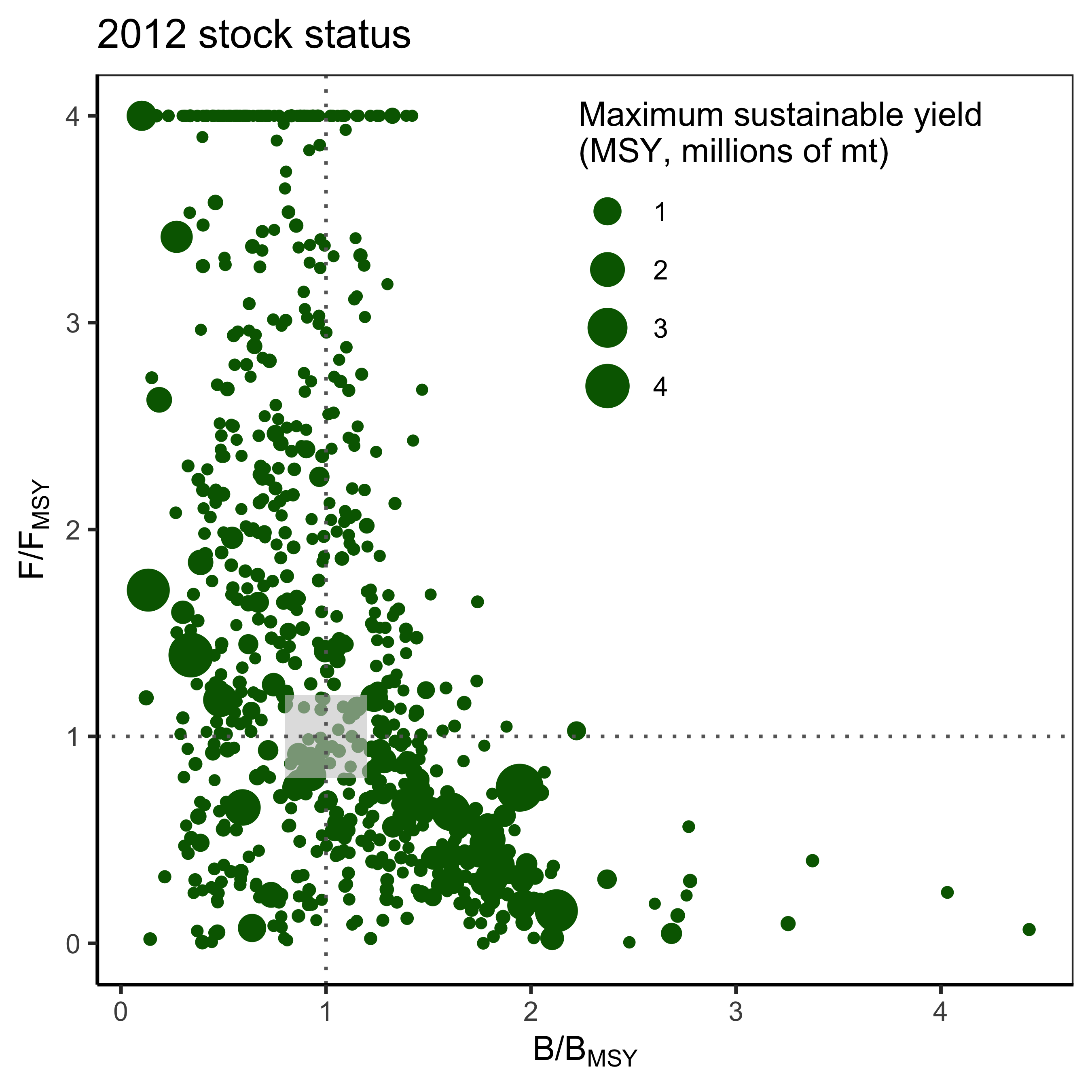

Supplement: S1 Fig — The transparent grey box indicates near optimal fisheries management (i.e., i.e., 0.8 ≤ B/BMSY ≤ 1.2 and 0.8 ≤ F/FMSY ≤ 1.2). (TIFF) [file pone.0224347.s002.tiff]

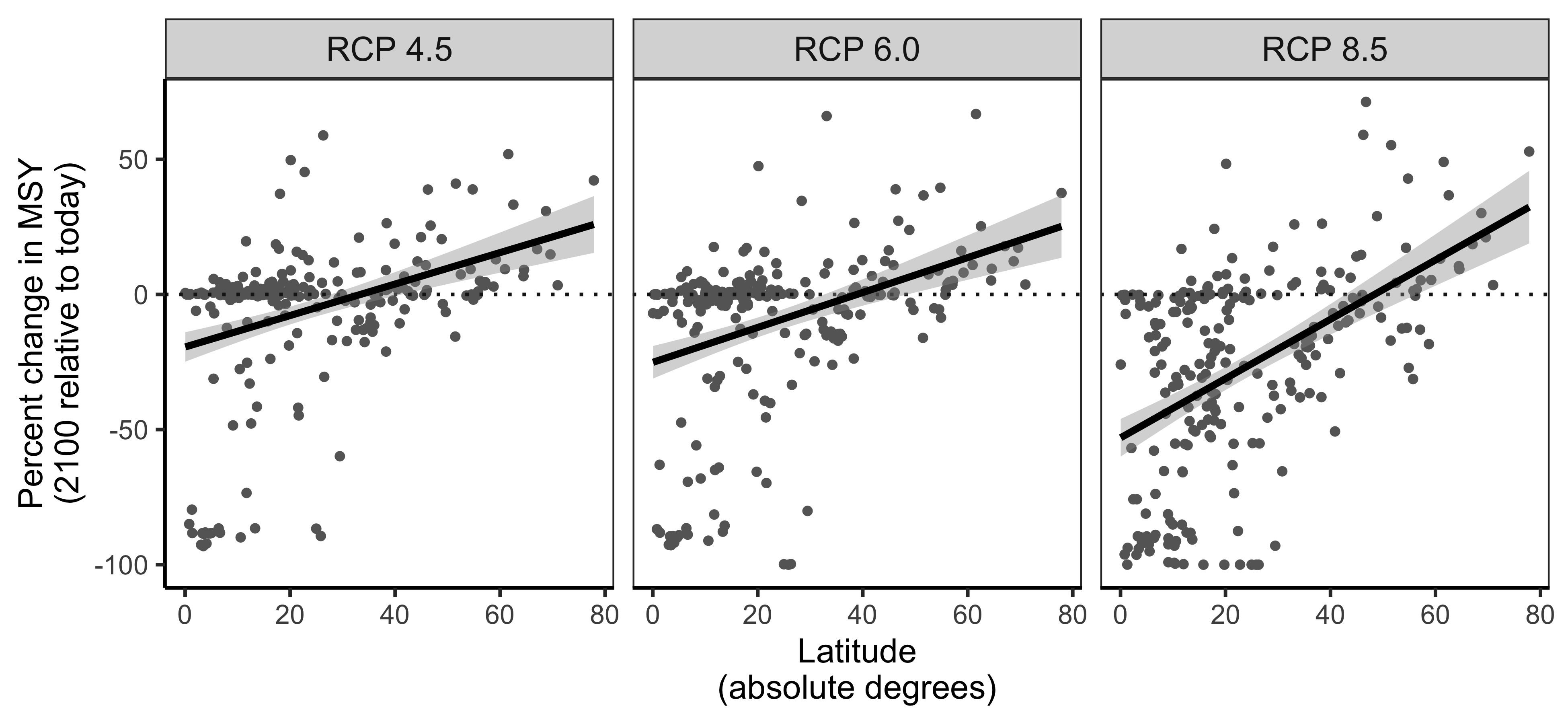

Supplement: S2 Fig — (TIFF) [file pone.0224347.s003.tiff]
